# TNRC6C Functions as a Tumor Suppressor and Is Frequently Downregulated in Papillary Thyroid Cancer

**DOI:** 10.1155/2021/6686998

**Published:** 2021-01-30

**Authors:** Zhenqin Cai, Tianyu Zhai, Dilidaer Muhanhali, Yan Ling

**Affiliations:** Department of Endocrinology and Metabolism, Zhongshan Hospital, Fudan University, No.180 Fenglin Road, Shanghai 200032, China

## Abstract

Our previous study found that trinucleotide repeat containing adaptor 6C (TNRC6C) may act as a tumor suppressor in papillary thyroid cancer (PTC). In this study, we aimed to confirm the effect of TNRC6C on PTC and investigate the underlying molecular mechanism. The difference of mRNA level of TNRC6C between PTC tissue and noncancerous thyroid tissue and the association of expression level of TNRC6C with clinicopathological features of PTC were analyzed using TCGA data. Immunohistochemical assay was performed to detect the protein expression of TNRC6C in PTC and its adjacent noncancerous tissue. Cell proliferation, migration, invasion, and apoptosis were analyzed after knockdown or overexpression of TNRC6C in BCPAP cells. RNA-sequencing was performed to find the target genes of TNRC6C, and potential targets were validated in BCPAP and TPC1 cells. Our results showed that TNRC6C was downregulated in PTC, and lower expression level of TNRC6C was associated with worse clinicopathological features. Overexpression of TNRC6C significantly inhibited proliferation, migration, and invasion of BCPAP cells and promoted its apoptosis, while knockdown of TNRC6C acted the opposite role. By analyzing RNA-sequencing data and TCGA data, 12 genes (SCD, CRLF1, APCDD1L, CTHRC1, PTPRU, ALDH1A3, VCAN, TNC, ECE1, COL1A1, CAMK2N2, and MMP14) were considered as potential target genes of TNRC6C, and most of them were associated with clinicopathological features of PTC in TCGA. All of them except CAMK2N2 were significantly downregulated after overexpressing TNRC6C. Our study demonstrated that TNRC6C functions as a tumor suppressor in PTC and may serve as a useful therapeutic target and prognostic marker for PTC patients.

## 1. Introduction

Thyroid cancer is the most common endocrine cancer, and a rapid increase in incidence and moderate increase in mortality of thyroid cancer were observed recently in China and worldwide [1, 2]. Among all thyroid cancers, papillary thyroid cancer (PTC) is the most common type, accounting for ∼80% of the cases [3, 4]. Although there is a relatively good prognosis of patients with PTC, 5–10% of patients experience local recurrence and distant metastasis [3]. Most of these advanced patients progress to resist radioiodine treatment, indicating a poor prognosis [3]. Hence, understanding the molecular mechanisms underlying the development and progression of PTC is essential.

Trinucleotide repeat containing 6 (TNRC6) proteins, including TNRC6A, TNRC6B, and TNRC6C, are important for miRNA-mediated gene silencing and serve scaffolding functions within miRNA-induced silencing complex [5]. Reduced expression of TNRC6 protein has been reported in gastric [6], colorectal [6], and non-small cell lung cancers [7]. In our previous study, we demonstrated that a long noncoding antisense RNA TNRC6C-AS1 promoted the malignant behavior of PTC and inhibited its intake of iodine by downregulating the expression of TNRC6C [8]. We found that the proliferation, migration, and invasion ability of TPC1 cells were weakened, and the proportion of apoptotic cells increased by overexpressing the TNRC6C, while knockdown of TNRC6C acted the opposite role [8]. Our findings suggest that TNRC6C participates in the development and progression of PTC.

As it has an important role in miRNA-induced posttranscriptional silencing pathway, we postulated that TNRC6C may be involved in the repression of some oncogenes, and reduced expression of TNRC6C may increase some miRNA-regulated oncogenes. In this study, we aimed to confirm the role of TNRC6C in PTC unequivocally by validating this hypothesis. First, we investigated the effect of TNRC6C on proliferation, apoptosis, migration, and invasion of another PTC cell line, the BCPAP cells. Second, we analyzed the expression of TNRC6C in PTC tissues and their adjacent normal thyroid tissues by immunohistochemical methods. We then analyzed the association of TNRC6C expression with clinicopathological features of PTC. To reveal the downstream targets of TNRC6C, we performed differential gene expression analysis using RNA-seq after overexpression of TNRC6C in BCPAP cells. Finally, we verified the downregulated targeted genes by quantitative RT-PCR after TNRC6C overexpression and investigated their associations with clinicopathological features of PTC.

## 2. Materials and Methods

### 2.1. Patients and Samples

We collected primary PTC tissues and their adjacent noncancerous tissues during surgery from 76 patients who presented to Zhongshan Hospital affiliated to Fudan University between December 2017 and December 2018. All patients were pathologically diagnosed with PTC, and fresh samples were frozen in liquid nitrogen. This study was approved by the Ethics Committee of Zhongshan Hospital. All enrolled patients provided written informed consent.

### 2.2. Data Acquisition and DEGs Identification from TCGA Database

All data including information on mRNA expression levels and clinical features of patients with PTC were downloaded from TCGA thyroid carcinoma cohort by the Genomic Data Commons (GDC) Data Transfer Tool, which contains 502 PTC and 58 adjacent noncancerous samples (https://tcgadata.nci.nih.gov/tcga). The differentially expressed genes (DEGs) between PTC tissue and noncancerous thyroid tissue were identified using edgeR package [9] according to the following criteria: (I)|log2FC|>1; (II)FDR<0.05. The association of expression levels of TNRC6C with clinicopathological features of PTC was analyzed.

### 2.3. Immunohistochemistry

Immunohistochemical (IHC) analysis was performed on formalin-fixed and paraffin-embedded PTC sections. Briefly, the sections were heated overnight at 56°C, deparaffinized by xylene, and rehydrated in a series of alcohol solutions. The TRIS-EDTA solution was used for antigen retrieval. After that, the sections were blocked in PBS containing 5% goat serum for 20 min, followed by overnight incubation with diluted primary antibody specific against TNRC6C (NOVUS, USA) at 4°C in a humidified chamber. On the next day, secondary antibody (Cell Signaling, USA) was added to the slides and incubated at 37°C for 30 minutes. After PBS washing, DAB (Dako, Denmark) was used for staining reaction. Finally, the sections were counterstained with 10% hematoxylin.

The TNRC6C levels were independently evaluated by two pathologists in a blinded fashion using a semiquantitative method by multiplying staining intensity by the percentage of positive staining cells (H-score). The tumor areas were divided into quarters, and scenes at five random microscopic fields were chosen from each quarter and the central area. Staining intensity was scored as follows: absent (0 point), weak (1 point), intermediate (2 points), and strong (3 points). The percentage of positive staining cells was scored as follows: 0∼5% (0 point), 6∼25% (1 point), 26∼50% (2 points), 51%∼75% (3 points), and 76∼100% (4 points). The TNRC6C expression levels were classified into four groups: negative (0 point), weakly positive (1–4 points), intermediately positive (5–8 points), and strongly positive (9–12 points). The patients with a final score of 0–4 points were defined as low TNRC6C expression, and those with a final score of 5–12 points were defined as high TNRC6C expression.

### 2.4. Cell Culture and Transfection

We purchased human PTC-derived cell lines (TPC1 and BCPAP) and normal thyroid epithelial cell line (Nthy-ori3-1) from the Cell Bank of Chinese Academy of Sciences (Shanghai, China). All cell lines were cultured in DMEM (HyClone, USA) containing 10% fetal bovine serum, streptomycin (100 mg/ml), and penicillin (100 U/ml) and incubated at 37°C in a humidified atmosphere containing 5% CO_2_.

We used Lipofectamine 2000 reagent (Invitrogen, USA) for siRNA or plasmid transfection following the manufacturer's instructions. TNRC6C-specific small interference RNAs (siRNAs) were designed and synthesized by Biosune Company (Shanghai, China). For overexpression of TNRC6C, pcDNA3.1-TNRC6C plasmid was constructed by cloning the full length of cDNA fragment of human TNRC6C into pcDNA3.1 plasmid (Invitrogen, USA). Transfection efficiency was evaluated by real-time qPCR at 48 hours after the transfection. We used cells transfected with control-siRNA or pcDNA3.1-empty plasmid as a negative control. All siRNA sequences can be found in Supplementary Table 1.

### 2.5. Real-Time qPCR Analysis and RNA-Sequencing

Total RNA was isolated using TRIzol method (Takara, Japan). RNA quality control was performed using NanoDrop2000 Spectrophotometer (Thermo Scientific, USA). The Reverse Transcription Kit (Takara, Japan) was used to perform reverse transcription. Quantitative PCR was performed using the SYBR Green assay (Takara, Japan) on the ABI7500 machine (Applied Biosystems, USA). *β*-Actin was selected as the internal reference gene. Results were analyzed by 2^−△△ Ct^ method. All primer sequences can be found in Supplementary Table 1.

Total RNA was isolated from three BCPAP cell samples transfected with pcDNA3.1-TNRC6C plasmid and three BCPAP cell samples transfected with pcDNA3.1-empty plasmid for RNA-sequencing. RNA-sequencing was done by the Beijing Genomics Institute (Shenzhen, China).

Briefly, oligo (dT) attached magnetic beads were used to purify mRNA. Purified mRNA was fragmented, reversely transcribed, and amplified by PCR. The double-stranded PCR products from the previous step were heated, denatured, and circularized by the splint oligo sequence to get the final library. The final library was amplified with phi29 to make DNA nanoball (DNB), which had more than 300 copies of one molecule, and DNBs were loaded into the patterned nanoarray, and pair-end 100 bases reads were generated on BGIseq500 platform.

### 2.6. Cell Proliferation Assay

Cell proliferation was determined by cell counting kit-8 (CCK-8) (Dojindo, Japan). BCPAP cells were transfected with TNRC6C-siRNA or pcDNA3.1-TNRC6C plasmid for 48h. Cell suspension was seeded into 96-well plates at the initial density of 5 × 10^3^ cells/well, and 10 *μ*l CCK-8 reagent was added to each well and incubated at 37°C for 4 h at various time points (0 h, 24 h, 48 h, and 72 h). Afterwards, Microplate Reader (RT6000, China) was used to measure the absorbance at 450 nm of each well.

### 2.7. Wound-Healing Assay

BCPAP cells were seeded at 2 × 10^5^/well into 6-well plates and transfected for 48 h. When cells reached 80–90% confluence, cell monolayers were scratched with a 20 *μ*l pipette tip and cultured in serum-free medium. Images at various time points (0 h, 24 h, and 48 h) were taken, and the relative migration area was determined by Image *J* software (National Institutes of Health, USA).

### 2.8. Flow Cytometry Assay

Transfected cells were harvested with EDTA-free trypsin and washed three times with phosphate-buffered saline (PBS). After that, cell apoptosis was measured using Cell Cycle and Apoptosis Analysis Kit (Beyotime, Shanghai, China) on a FACSCalibur flow cytometry (BD Biosciences, USA). The proportion of early apoptotic cells and late apoptotic cells are added to get the percentage of apoptotic cells.

### 2.9. Transwell Invasion and Migration Assay

Transwell invasion and migration assay were performed using 8.0 *μ*m Corning chambers (Corning, USA), and the chambers were matrix-coated in invasion assay (BD Biosciences, USA). Transfected cell suspension was prepared in 200 *μ*l serum-free medium and added into the top chambers, while 600 *μ*l medium containing 10% FBS was added into the bottom chambers. After incubation at 37°C for various periods (24 h and 36 h), cells were fixed in paraformaldehyde (4%) for 30 min and stained in crystal violet (0.1%) for 20 min. A cotton swab was used to remove cells on the top surface. Images at five randomly microscopic fields were taken, and the cell number was quantified by ImageJ software (National Institutes of Health, USA).

### 2.10. Statistical Analysis

All the experiments were independently repeated three times, and the data was presented as mean ± standard deviation (SD). We used SPSS 20.0 (IBM, USA) to perform the statistical analysis. Mann-Whitney and Wilcoxon tests were used to compare expression levels of TNRC6C in PTC tissues with noncancerous tissues. Chi-square test was used to analyze the association of expression levels of genes with clinicopathological features of PTC. Independent *t*-test was used to compare the difference between cell groups. Statistical significance was established at *p* < 0.05.

## 3. Results

### 3.1. TNRC6C Is Downregulated in PTC

**Figure 1:**
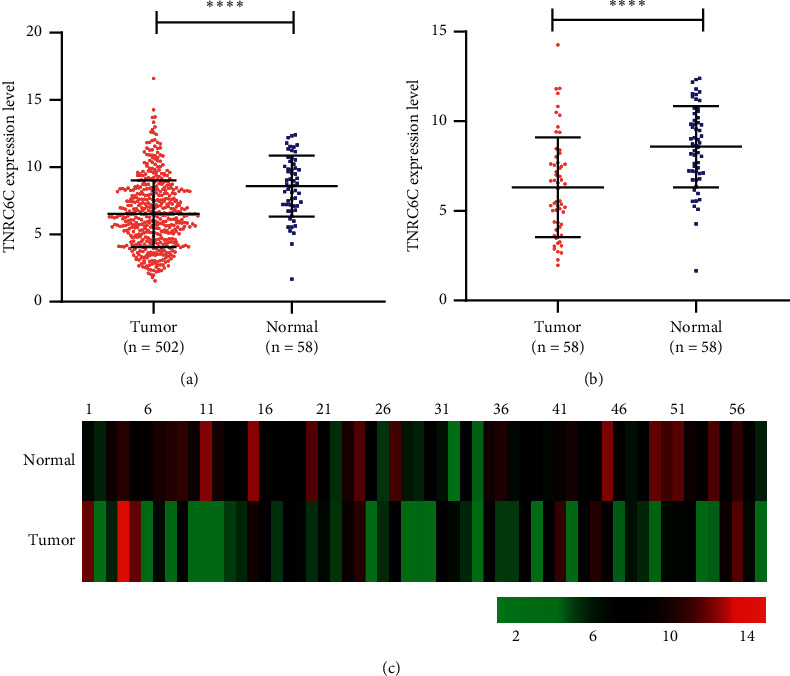
Differential mRNA expression of TNRC6C in PTCs and noncancerous thyroid tissues in TCGA database. (a) TNRC6C mRNA expression was significantly downregulated in PTC tissues (*n* = 502) compared with the noncancerous tissues (*n* = 58). (b) TNRC6C mRNA expression was significantly downregulated in 58 paired PTC tissues and their adjacent noncancerous tissues. (c) Heatmap of TNRC6C mRNA expression in 58 paired PTC tissues and their adjacent noncancerous tissues. TNRC6C mRNA expression was frequently downregulated in PTC tissues. ^*∗∗∗∗*^*P* < 0.0001.

**Figure 2:**
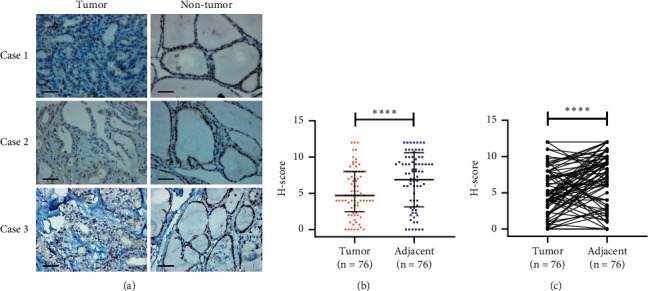
Differential protein expression of TNRC6C in PTCs and corresponding adjacent noncancerous tissues by immunohistochemical analysis. (a) Representative IHC images of three paired PTC tissues and adjacent noncancerous tissues. Scale bars indicate 200 um. (b) Scatter plot of TNRC6C protein expression presented as H-score in PTC tissues and adjacent noncancerous tissues. (c) Before–after graph of TNRC6C protein expression presented as H-score in PTC tissues and adjacent noncancerous tissues.

By analyzing the TCGA data, we found that TNRC6C was downregulated in 502 PTC samples compared with 58 noncancerous samples (Figure 1(a)). The TNRC6C was also downregulated in PTC samples compared with their adjacent noncancerous samples in a total of 58 paired samples in TCGA (Figures 1(b) and 1(c)). In addition, we conducted IHC assay of TNRC6C in 76 paired PTC samples and their adjacent noncancerous samples from patients admitted to Zhongshan Hospital affiliated to Fudan University. We found that higher staining percentage and intensity of TNRC6C commonly occurred in adjacent noncancerous tissues but was rare in PTC tissues (Figure 2(a); Table 1). Quantitation of the IHC staining also showed higher TNRC6C expression in adjacent noncancerous tissues than that in PTC tissues (Figures 2(b) and 2(c)). These results indicated that TNRC6C was frequently downregulated in PTC.

### 3.2. Lower Expression Level of TNRC6C Is Associated with Worse Clinicopathological Features of PTC

We analyzed the association between TNRC6C expression and clinicopathological features of PTC using TCGA data. A total of 502 PTCs were divided into TNRC6C low expression group and TNRC6C high expression group based on the median TNRC6C expression value, and we compared the clinicopathological features between the two groups (Table 2). Strong associations were observed between TNRC6C expression and age (*P*=0.004), *T* classification (*P*=0.005), N classification (*P*=0.014), clinical stage (*P*=0.002), and histological type (*P* < 0.001). These results indicated that lower expression level of TNRC6C was associated with a larger tumor size, lymph node metastasis, advanced clinical stage, and more aggressive histological type.

### 3.3. TNRC6C Modulates Proliferation, Migration, Invasion, and Apoptosis of PTC Cells

In our previous studies, we found that overexpression of TNRC6C in TPC1 cells significantly inhibited cell proliferation, cell migration, and invasion abilities and promoted cell apoptosis, while the opposite was observed by downregulation of TNRC6C [8]. In this study, we further confirmed the effect of TNRC6C on the progression of PTC through manipulating TNRC6C expression in BCPAP cells.

**Figure 3:**
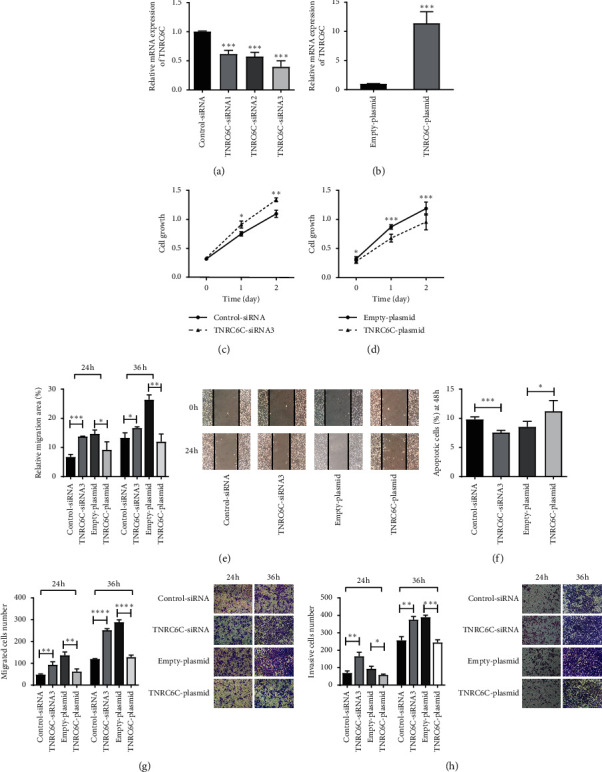
TNRC6C regulates the proliferation, apoptosis, migration, and invasion abilities of BCPAP cells. (a) BCPAP cells were transfected with TNRC6C-siRNA1, TNRC6C-siRNA2, TNRC6C-siRNA3, and control-SiRNA, respectively. The relative mRNA expression of TNRC6C was quantified by real-time qPCR. (b) BCPAP cells were transfected with pcDNA3.1-TNRC6C plasmid and pcDNA3.1-empty plasmid, respectively. The relative mRNA expression of TNRC6C was quantified by real-time qPCR. (c) Growth curve of BCPAP cells determined by CCK8 assay after transfection with TNRC6C-SiRNA3 or control-SiRNA. (d) Growth curve of BCPAP cells determined by CCK8 assays after transfection with pcDNA3.1-TNRC6C plasmid or pcDNA3.1-empty plasmid. (e) The effect of TNRC6C knockdown or overexpression on the migration of BCPAP cells was assessed using wound-healing assay. Quantitative analysis (left) and representative images (right). (f) The effect of TNRC6C knockdown or overexpression on BCPAP cell apoptosis was determined by flow cytometry assay. (g) The effect of TNRC6C knockdown or overexpression on the migration of BCPAP cells was assessed using transwell migration assay. Quantitative analysis (left) and representative images (right). (h) The effect of TNRC6C knockdown or overexpression on the invasion of BCPAP cells was assessed using transwell invasion assays (quantitative analysis (left) and representative images (right). Values represent the mean ± SD from three independent experiments; empty-plasmid, pcDNA3.1-empty plasmid; TNRC6C-plasmid, pcDNA3.1-TNRC6C plasmid; ^*∗*^*P* < 0.05; ^*∗∗*^*P* < 0.01; ^*∗∗∗*^*P* < 0.001; ^*∗∗∗∗*^*P* < 0.0001.

First, we knocked down TNRC6C via transfecting cells with TNRC6C-specific siRNA. We performed qPCR to confirm the knockdown efficiency and chose TNRC6C-siRNA3 for further experiments (Figure 3(a)). Downregulation of TNRC6C in BCPAP cells significantly promoted cell proliferation and inhibited cell apoptosis (Figures 3(c) and 3(f)). In addition, downregulation of TNRC6C also enhanced cell migration and invasion abilities (Figures 3(e), 3(g), and 3(h)). We also overexpressed TNRC6C via transfecting cells with pcDNA3.1-TNRC6C plasmid. The mRNA level of TNRC6C was 10 times higher than that in cells transfected with pcDNA3.1-empty plasmid (Figure 3(b)). Overexpression of TNRC6C significantly inhibited the aggressiveness of BCPAP cells (Figures 3(d), 3(e), 3(g), and 3(h)) and promoted cell apoptosis (Figures 3(f)). These results indicated that TNRC6C might function as a tumor suppressor in PTC.

### 3.4. Identification of Potential Target Genes of TNRC6C

**Figure 4:**
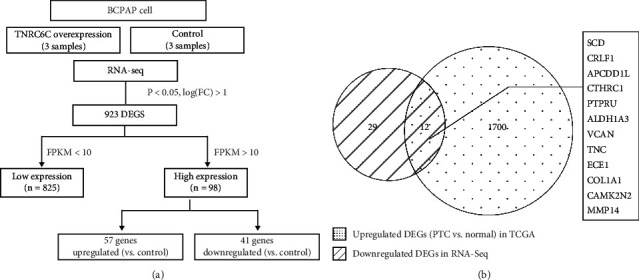
Identification of potential target genes of TNRC6C using RNA-seq. (a) 923 differential gene were identified by differential gene expression analysis in TNRC6C overexpressed BCPAP cells compared with control cells. (b) Venn chart of the DEGs downregulated by TNRC6C overexpression in RNA-seq and DEGs upregulated in PTC compared with noncancerous tissues in TCGA datasets. The DEG was defined as *P* < 0.05, |Log_2_FC|>1.

After demonstrating the tumor suppressor role of TNRC6C on PTC, we further screened for potential target genes of TNRC6C. First, we performed RNA sequencing in TNRC6C overexpression BCPAP cells and control cells. By using the RNA-sequencing data, we identified 923 DEGs (*P* < 0.05, |log_2_(fold change)|>1) between TNRC6C overexpression BCPAP cells and control cells through differential expression gene (DEGs) analysis. Among them, 825 genes were defined as low expression (FPKM<10), and 98 genes were defined as high expression (FPKM>10). Given that TNRC6C played a tumor suppressor role, we speculated that TNRC6C might inhibit the development and progression of PTC through downregulating some oncogenic genes. Therefore, we focused on genes negatively regulated by TNRC6C, especially those with high expression levels (Figure 4(a)). Next, we performed differential expression gene analysis using TCGA data and identified 1713 DEGs (*P* < 0.05, |log_2_(fold change)|>1) with higher expression levels in PTC tissues compared with noncancerous tissues. Twelve genes, which were both downregulated by TNRC6C and upregulated in PTC, were considered as potential target genes of TNRC6C (Figure 4(b)). These genes are listed in descending order of |log_2_(fold change)| value between TNRC6C overexpressed BCPAP cells and control cells (Table 3).

### 3.5. Overexpression of TNRC6C Downregulated the Expression of Potential Target Genes at the mRNA Level

**Figure 5:**
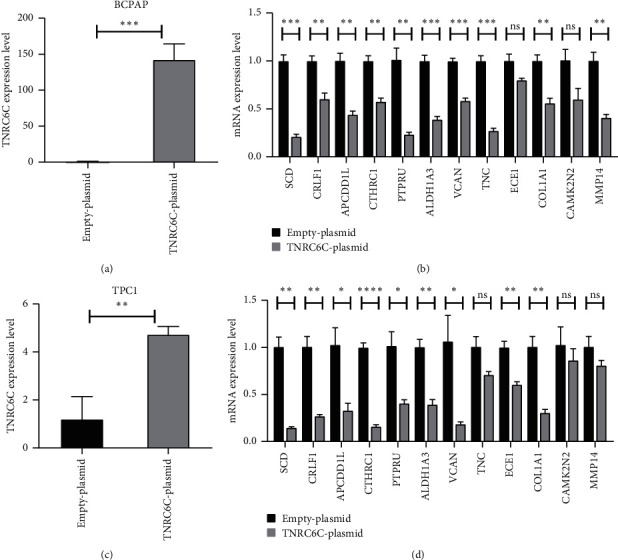
Overexpression of TNRC6C downregulates the expression of potential target genes at the mRNA level in BCPAP and TPC1 cells. (a) The relative mRNA level of TNRC6C in TNRC6C overexpressed BCPAP cells and control BCPAP cells. (b) The relative mRNA levels of potential target genes in TNRC6C overexpressed BCPAP cells and control BCPAP cells. (c) The relative mRNA level of TNRC6C in TNRC6C overexpressed TPC1 cells and control TPC1 cells. (d) The relative mRNA levels of potential target genes in TNRC6C overexpressed TPC1 cells and control TPC1 cells. ^*∗*^*P* < 0.05; ^*∗∗*^*P* < 0.01; ^*∗∗∗*^*P* < 0.001; ^*∗∗∗∗*^*P* < 0.0001; ns, not significant.

We validated the negative regulation of TNRC6C on 12 potential target genes through qPCR. We overexpressed TNRC6C in both BCPAP and TPC1 cells via transfecting cells with pcDNA3.1-TNRC6C plasmid. Compared with control cells, the mRNA level of TNRC6C was more than 100-fold higher in TNRC6C overexpressed BCPAP cells (Figure 5(a)) and more than fourfold higher in TNRC6C overexpressed TPC1 cells (Figure 5(c)). All tested genes were significantly downregulated in BCPAP cells after overexpressing TNRC6C except ECE1 and CAMK2N2. In TPC1 cells, all tested genes were significantly downregulated after overexpressing TNRC6C except TNC, CAMK2N2, and MMP14 (Figures 5(b) and 5(d)).

### 3.6. Higher Expression Levels of Potential Target Genes Were Associated with Worse Clinicopathological Features of PTC

We analyzed the relationship between the expression levels of 12 potential target genes and the clinicopathological features of PTC using TCGA data (Table 4). Higher expression levels of genes were associated with lymph node metastasis and histological type. We found that higher expression levels of some genes were also associated with larger tumor size and advanced clinical stage such as collagen triple helix repeat containing 1 (CTHRC1), APC downregulated 1 like (APCDD1L), collagen type I alpha 1 chain (COL1A1), versican (VCAN), tenascin-C (TNC), and matrix metalloproteases 14 (MMP14). These genes were more likely to be the mediators through which TNRC6C played its tumor suppressor role.

## 4. Discussion

In this study, we found that overexpression of TNRC6C significantly suppressed BCPAP cell proliferation, promoted its apoptosis, and decreased its migration and invasion abilities, while knockdown of TNRC6C acted the opposite role. These findings were consistent with our previous observations in TPC1 cells [8]. The in vitro results suggested that TNRC6C has an inhibiting effect on the malignant behavior of PTC cells. The low expression level of TNRC6C in PTC tissues also indicated that the defect of TNRC6C may take part in the development of PTC. Further, we found that higher TNRC6C expression levels were associated with less aggressive clinicopathological features of PTC, indicating a better prognosis of PTC patients with a higher expression of TNRC6C compared with those with lower expression levels. Based on the aforementioned findings, we considered that TNRC6C may act as a tumor suppressor in PTC.

Current evidence shows that TNRC6C functions as an important component within miRNA-induced silencing complex [10–14]. TNRC6C may take part in the repression of some miRNA-regulated oncogenes, which contribute to the development of PTC. Of course, it is not known whether TNRC6C functions through other mechanisms besides its scaffolding function within miRNA-induced silencing complex. Our IHC results showed that TNR6C6 was primarily localized in the nucleus, indicating that it may also regulate genes expression at the transcriptional level. Regardless of the kind of mechanism, our findings support the tumor suppression role of TNRC6C in PTC, and it is important to find out the oncogenes targeted by it.

High-throughput transcriptome sequencing (RNA-seq) is an important method for discovering differentially expressed genes under different conditions and therefore is commonly used to elucidate the regulatory relationship between the molecules. After overexpressing TNRC6C in BCPAP cells, differential gene expression was analyzed using RNA-seq data to find out the downstream targets of TNRC6C. We focused on the downregulated genes after TNRC6C overexpression, as they have the potential of being the targeted oncogenes. Twelve DEGs were significantly downregulated after TNRC6C overexpression and upregulated in PTC compared with noncancerous tissues in TCGA datasets. Among these 12 DEGs of interest, higher expression levels of CTHRC1, APCDD1L, COL1A1, VCAN, TNC, and MMP14 were significantly associated with larger tumor size, cervical lymph node metastasis, advanced clinical stage, and aggressive histological type of PTC.

The association of CTHRC1 with tumor progression is demonstrated in various cancers, including melanoma, hepatocellular carcinoma, lung cancer, gastric cancer, pancreatic cancer, breast cancer, and colorectal cancer [15–17]. Different mechanisms for its role of promoting tumor progression were assumed for various cancers. In a recent study, CTHRC1 serves as a prometastatic gene of non-small cell lung cancer, and the invasion and metastasis ability mediated by it were MMP7- and MMP9-dependent [16]. The role of CTHRC1 in thyroid cancers is not clear. A few studies found that there was an aberrant expression of CTHRC1 in thyroid cancers [15], and CTHRC1 expression levels in PTC were significantly correlated with lymph node metastases, the expression of E-cadherin and vimentin [18]. COL1A1 is the *α*1 chain of type I collagen, and COL1A1 is aberrantly expressed in varieties of cancers, suggesting it may serve as an important diagnostic and prognostic marker and potential therapeutic target [19–22]. In vitro studies have shown that downregulation of COL1A1 in a PTC cell line (TPC-1) inhibited cell proliferation, invasion, and migration [23, 24]. VCAN is a large proteoglycan in extracellular matrix. In addition to cell adhesion and tissue morphogenesis, it is also implicated in tumorigenesis and tumor progression [25]. Increased expression of VCAN has been reported in several cancers and is associated with adverse outcomes [22, 26–28]. A few studies have shown that the expression of VCAN was upregulated in thyroid cancer tissues, and it was associated with malignant behavior of thyroid carcinoma [29, 30]. TNC is an extracellular matrix glycoprotein and is typically lowly expressed in adult tissue [31]. However, its expression is increased in disease undergoing tissue remodeling, such as cancers [31]. In the tumor stroma of most epithelial malignancies, there is an increased deposition of TNC, which is associated with a poor prognosis [32–34]. Reexpression of TNC has also been observed in papillary and medullary thyroid carcinomas [35, 36]. MMP14 is a membrane-type matrix metalloproteinase, which mediates processes such as extracellular matrix degradation and remodeling, cell invasion, and cancer metastasis [37]. It has been shown that MMP14 takes part in epithelial-mesenchymal transition and promoted the progression of various cancers, such as hepatocellular carcinoma, breast cancer, glioma, and sarcoma [38–41]. The expression of MMP14 was found significantly increased in thyroid tumor cell lines and PTC samples [42,43] and was associated with the invasiveness of thyroid cancer [44]. APCDD1L was found to be associated with aortic diseases [45] and smoking-related DNA methylation in nucleus accumbens [46], but its effect on cancers was never reported.

In conclusion, we demonstrated that TNRC6C functions as a tumor suppressor and is frequently downregulated in PTC. We also searched for the downstream target genes of TNRC6C in this study. Potential targets of TNRC6C, such as CTHRC1, APCDD1L, COL1A1, VCAN, TNC, and MMP14, may play important roles in the development and progression of PTC. TNRC6C may serve as a useful therapeutic target and prognostic marker for PTC patients.

## Figures and Tables

**Table 1:** Differential protein expression of TNRC6C in PTCs and corresponding adjacent noncancerous tissues.

	PTC (*n* = 78)	Adjacent tissues (*n* = 78)	*P* value
*Four levels*			
Negative	6	6	0.002
Weakly positive	29	16	
Moderately positive	28	20	
Strongly positive	13	34	

*Two levels*			
Low expression	35	22	0.029
High expression	41	54	

**Table 2:** The association of TNRC6C expression with clinicopathological features of PTC in TCGA database.

Clinicopathological features	TNRC6C expression level		*P* value
	Low	High	
*Age*			
≤46	110	142	0.004
>46	141	109	

*Gender*			
Female	191	176	0.131
Male	60	75	

*T classification*			
T1	67	76	0.002
T2	68	96	
T3	98	72	
T4	17	6	

*N classification*			
N0	101	128	0.014
N1	124	99	

*M classification*			
M0	136	146	0.178
M1	2	7	

*Clinical stage*			
I	127	154	0.005
II	22	30	
III	66	46	
IV	36	19	

*Histological type*			
Classical/follicular	213	244	<0.001
Tall cell	32	4	

*Focality*			
Unifocal	140	126	0.243
Multifocal	107	119	

Age and TNRC6C expression level were divided into two groups according to the median.

**Table 3:** Significant fold changes of 12 potential target genes expression between TNRC6C overexpressed BCPAP cells and control cells.

Gene symbol	Log2(FC)	*P* value
SCD	−1.756	<0.001
CRLF1	−1.655	<0.001
APCDD1L	−1.445	<0.001
CTHRC1	−1.299	<0.001
PTPRU	−1.241	<0.001
ALDH1A3	−1.236	<0.001
VCAN	−1.124	<0.001
TNC	−1.091	<0.001
ECE1	−1.064	<0.001
COL1A1	−1.034	<0.001
CAMK2N2	−1.003	<0.001
MMP14	−1.000	<0.001

**Table 4:** The association between potential target genes and clinicopathological features of PTC.

Gene symbol	T classification	N classification	Clinical stage	Histological type
SCD	ns	<0.001	ns	ns
CRLF1	ns	<0.001	ns	0.015
APCDD1L	0.001	<0.001	0.006	<0.001
CTHRC1	0.008	<0.001	0.002	<0.001
PTPRU	0.035	<0.001	ns	0.005
ALDH1A3	ns	<0.001	ns	0.006
VCAN	<0.001	<0.001	<0.001	<0.001
TNC	<0.001	<0.001	0.011	<0.001
ECE1	ns	<0.001	ns	ns
COL1A1	<0.001	<0.001	<0.001	<0.001
CAMK2N2	ns	ns	ns	ns
MMP14	0.008	<0.001	0.047	0.002

Expression levels of genes were divided into two groups according to the median. The associations of expression levels of genes with clinicopathological features of PTC were analyzed using chi-square test. The significant results indicated that higher expression levels were associated with worse clinicopathological features of PTC.

## Data Availability

The data used to support the findings of this study are included within the article or are available from the corresponding author upon request.
