# Supplementary material for: TNRC6C Functions as a Tumor Suppressor and Is Frequently Downregulated in Papillary Thyroid Cancer
Source: Int J Endocrinol. 2021 Jan 30;2021:6686998. doi: 10.1155/2021/6686998 (PMC7867448; doi:10.1155/2021/6686998)
Supplement: Supplementary Materials — All primer and siRNA sequences can be found in Supplementary Table 1. [file 6686998.f1.docx]

**Supplementary Table S1** Primer and siRNA sequences for this study

| **Gene** | **Forward Primer** | **Reverse Primer** |
| --- | --- | --- |
|  | **(sense)** | **(anti-sense)** |
| **β-actin** | TCACCCACACTGTGCCCATCTACGA | CAGCGGAACCGCTCATTGCCAATGG |
| **TNRC6C** | AATGGACACTATGATTGGAGATGG | CCTATGGCACTGACTGGACTG |
| **SCD** | TTCTTGCGATATGCTGTGGTG | AGTAGTCATAGGGAAAGGAGTGG |
| **CRLF1** | CTGGAAGGTGGTGGACGATG | GGAGCCATAGATGCCAAAGGG |
| **CTHRC1** | GTCAGCGTTGGTATTTCACATTC | CCTTCACAAAGTCCTTCCACAG |
| **PTPRU** | GGGCTTATCCTGGGCATCTG | GTGTCTTCTCCTGGCGGTAG |
| **ALDH1A3** | CTCTTCATCAAACCCACTGTCTTC | GGCTGCTGTGAGTCCATAGTC |
| **TNC** | CTGAGGTTGGCTGGGATGG | AGATGGAGACTGTATAAGGCGTAG |
| **COL1A1** | GGTGAACCTGGTGCTCCTG | CCTCGCTTTCCTTCCTCTCC |
| **APCDD1L** | TGGTGTTTGAGGTCACACGG | TCCATCTTGAAAAGCTCGTACTC |
| **CAMK2N2** | CCTGCCCTACAGCGAAGAC | GCGAAGAAGGAGTTGGTGTC |
| **ECE1** | TCTCCACTCTCATCAACACCAC | AACTTCTCATCGGCGTCCTG |
| **MMP14** | TTCTGGCGGGTGAGGAATAAC | TCTCGTAGGCAGTGTTGATGG |
| **VCAN** | AGCGGAGACCAGTGTGAAC | AACATAACTTGGAAGGCAGAGG |
| **TNRC6C-siRNA1** | GCUGGGAUAAAGUGAUAAUUU | UUGACCCUAUUUCACUAUUAU |
| **TNRC6C-siRNA2** | CUCCAAUGCUGGCAUUAAUUU | UUGGCGAGAUAAAGGGAUUAU |
| **TNRC6C-siRNA3** | GCGAGAUAAAGGGAUUAUAUU | UUGCGGGAAGGAAAUCGAUUA |
